# Digital transformation in college libraries: The effect of digital reading on reader service satisfaction

**DOI:** 10.1371/journal.pone.0307699

**Published:** 2024-08-22

**Authors:** Yixin Lu, Shengguang Lin

**Affiliations:** 1 Library, Wenzhou University, Wenzhou, China; 2 School of Economics and Management, Wenzhou University of Technology, Wenzhou, China; Shahid Beheshti University of Medical Sciences, ISLAMIC REPUBLIC OF IRAN

## Abstract

In the pursuit of digital transformation, college libraries have increasingly embraced the promotion of digital reading as a critical initiative. While numerous studies have delved into the strategies employed by college libraries in their digital transformation endeavors, there remains a lack of research elucidating the direct influence of digital reading on reader service satisfaction within these institutions. Drawing upon the service quality model, this paper aims to address this gap by examining the multifaceted influence of digital reading on reader service satisfaction in college libraries. By examining the various dimensions of digital reading services, this study employs the fsQCA approach to uncover specific combinations that contribute to heightened levels of reader service satisfaction. The results reveal three distinct configurations that can explain the high level of reader service satisfaction. By elucidating these critical relationships, this research not only provides a contribution to the research regarding the evolving role of college libraries but also provides practical insights for college libraries aspiring to realize digital transformation by promoting digital reading.

## 1. Introduction

With the development of the digital era, digital transformation has become an indispensable step in the development of college libraries [1]. Digital transformation refers to the integration of digital technologies, processes, and strategies to revolutionize organizational operations and value delivery. In college libraries, it entails leveraging digital resources and services to enhance information access and usability, thereby enriching user experiences and adapting to evolving information landscapes. As an important way to realize digital transformation, digital reading not only adapts to the actual reading needs of current college students but also helps to improve reader service satisfaction. The most recent data show that 87% of Chinese university libraries have implemented digital reading programs to meet this digital change and to meet the evolving needs of the student population [2]. Meanwhile, in the United States, 92% of colleges have actively promoted digital reading practices within their library systems [3]. This surge in adoption underscores the global recognition of digital reading as a catalyst for improving the digital transformation of college libraries.

Existing studies have explored digital reading in college libraries from two perspectives. On the one hand, they investigate the impact of digital reading on learning and user experience. Research in this domain has revealed how digital resources can enhance student learning and make learning more interactive, accessible, and engaging [4, 5]. On the other hand, they provide an in-depth study of strategies to promote digital reading in the college library environment. These strategies include purchasing e-books, developing user-friendly digital interfaces, and integrating digital reading into the courses [1, 6]. These studies emphasize the potential benefits of digital reading but do not reveal the connection between digital reading and reader service satisfaction.

While previous literature reveals the effect of digital reading and promotional practices for digital reading, the direct impact of digital reading on reader service satisfaction is ignored. This question is noteworthy for two reasons. First, reader service satisfaction is a key indicator for evaluating the overall performance of the college library [7]. Understanding the impact of digital reading on reader satisfaction can help libraries identify the key measures to enhance their services. Second, understanding the unique impact of digital reading on reader service satisfaction can provide libraries with a strategic advantage and help them realize digital transformation as soon as possible.

To bridge this gap, this study conducts empirical research to reveal the intricate interactions between the various dimensions of digital reading services and their impact on reader service satisfaction within college libraries. First, we categorize the dimensions of digital reading services based on the service quality model [8]. Second, we employ the Fuzzy-Set Qualitative Comparative Analysis (fsQCA) model [9], a robust methodology for examining complex causal relationships, to explore how different dimensions of digital reading services interact with one another and jointly influence reader service satisfaction. Third, we reveal how specific dimensions of digital reading services (access and usability, resource consistency, timely assistance, credible services, and user support) combine as causal configurations to explain reader service satisfaction in college libraries.

This study makes a few contributions to the evolving role of college libraries. First, we unveil the direct impact of digital reading, which is important but frequently ignored in college library services. Second, this research identifies specific combinations of factors that contribute to reader service satisfaction and thus provides practical insights for libraries to improve their digital reading service. Third, this study reveals the multifaceted relationship between digital transformation and reader service satisfaction, thus promoting the ability of university libraries to better serve the changing needs of students in realizing digital transformation.

The structure of this paper is as follows. Section 2 describes related works and summarizes existing research on digital reading in college libraries. Section 3 gives an in-depth discussion of the impact of the complex interactions between different dimensions of digital reading services on reader service satisfaction in college libraries. Section 4 presents research data and describes research method. Section 5 describes the research results of the fsQCA analysis, offering insights into the configurations of digital reading service dimensions that contribute to reader service satisfaction. Section 6 concludes our study by summarizing findings, and discussing implications and limitations.

## 2. Literature review

### 2.1 Digital reading in college libraries

The emergence of digital technology has significantly changed the landscape of college libraries, and digital reading has become a core part of this transformation. Digital reading involves the use of electronic devices to access and engage with digital resources, such as e-books and academic journals [10]. Such a shift from traditional print materials to digital formats is driven by several factors, including the rising prevalence of digital resources, the convenience of remote access, and the environmental benefits of reduced paper use [11]. As a result, college libraries across the world have made significant investments in digital reading infrastructures (i.e. electronic library collections and user-friendly digital interfaces) to meet the changing needs of readers.

Although there is a significant growth in the adoption of digital reading in college libraries, research undertaken in this domain focuses primarily on its impact on learning outcomes and user experience. Some studies explored how digital reading can enhance library services by enabling interactive and immersive learning environments [12, 13]. In addition, studies examined relevant factors that affect the user experience of digital reading interfaces, such as usability, accessibility, and content relevance [14]. For instance, Xie (2022) investigated students’ user experiences when using the digital reading platform of a college library. This study revealed various aspects of the user experience, including the usability of the digital interface and the accessibility of digital resources.

In examining the interplay between various factors influencing satisfaction within digital reading services, it becomes evident that the effectiveness of these services is not isolated but rather interconnected with multiple dimensions. Our investigation draws attention to the intricate relationships between access and usability, resource consistency, timely assistance, credible services, and user support. For instance, the seamless integration of user support with access and usability significantly enhances the overall reader experience, as evidenced by studies conducted by Naumann [15]. Moreover, the credibility of digital services is underscored when there is consistency in the availability of resources, as highlighted by the work of Zhang et al. [16]. This dynamic interplay illuminates the importance of a holistic approach to digital reading services, emphasizing the need for college libraries to consider the synergies between these factors.

However, there remains a research gap regarding the relationship between digital reading in college libraries and reader service satisfaction. The neglect of this gap would limit our understanding of how the adoption of digital reading services affects the quality of overall library services and user satisfaction. Therefore, this paper seeks to bridge this gap by exploring how digital reading, as a core component of library services, directly contributes to reader service satisfaction, thereby providing valuable insights for college libraries aiming to optimize their digital reading services.

### 2.2 Reader service satisfaction

Reader service satisfaction is a fundamental element in evaluating the performance of library services in colleges. It pertains to the degree of satisfaction readers experience with the services, resources, and assistance provided by a college library [17]. A high level of reader service satisfaction indicates that the library can provide good reading conditions, enhance readers’ reading experience, and meet their different needs. Libraries with a high level of reader service satisfaction not only provide a broad range of collection resources, but also offer their readers easy access, and guidance on their services, and create a conducive environment for learning and reading [18].

Research on reader service satisfaction focuses on the factors that affect reader service satisfaction, including the accessibility of resources, the helpfulness of library staff, and the availability of digital services. Several studies have examined the relationship between different library services and reader service satisfaction. For instance, research revealed the importance of user-friendly digital interfaces and timely assistance in resolving questions related to digital reading [19]. Additionally, the credibility and reliability of digital resources are also identified as important factors in shaping readers’ perceptions of library services [20]. In recent empirical studies, researchers have delved into the intricate relationship between digital reading services and overall reader service satisfaction in college libraries. Notably, the work of Xie et al. [5] explored the impact of enhanced access and usability of digital resources, revealing a significant positive correlation with heightened levels of reader satisfaction. Furthermore, Li et al. [21] pointed out that there is a clear correlation between the consistency of digital resources, the timeliness and interactivity of assistance and readers’ overall service satisfaction. These empirical findings underscore the multifaceted nature of digital reading services and their pivotal role in shaping reader service satisfaction.

However, there is an important research gap regarding a comprehensive understanding of how the various dimensions of digital reading services interact with each other to collectively affect readers’ service satisfaction. This gap highlights the need for a comprehensive study of the interplay between the intricacies of digital reading and readers’ service satisfaction within the context of college libraries. Revealing this important issue will provide valuable insights into the evolving role of digital reading in shaping readers’ service satisfaction, which will inform strategic decision-making in libraries in the digital era.

## 3. Theoretical framework based on the service quality model

The service quality model was introduced as a foundational framework for understanding and assessing service quality across various industries [8]. This model posits that service quality is influenced by customer perceptions, which are shaped by comparing customers’ expectations of a service with their experience [22]. There are five dimensions identified by the service quality model that shape service quality: tangibility, reliability, responsiveness, assurance, and empathy. These dimensions provide a comprehensive framework for assessing service quality and are widely used in numerous service-related studies.

Drawing on the service quality model, we seek to develop a theoretical framework that relates its dimensions to digital reading services in college libraries. In this regard, we adapted and extended the service quality model to address the unique issues of digital reading. Specifically, the tangible dimensions include the physical and virtual elements that users encounter in digital reading, including the accessibility and usability of digital reading platforms. The tangible dimension also extends to the appearance and function of digital interfaces to ensure that they are user-friendly and intuitive to use. The reliability dimension relates to the consistency of the digital resources in the digital reading service. This dimension emphasizes resources are always available, perform reliably, and are free of technical problems to ensure a good reading experience. In terms of responsiveness, timely assistance in solving digital reading problems is an important aspect of the responsiveness of the digital reading service. This dimension examines whether the service can provide timely and effective support to users who encounter difficulties or need guidance, thus improving the overall quality of the service.

The assurance dimension relates to the trustworthiness of the digital reading services of the library. This dimension includes the accuracy and authenticity of digital resources as well as the reliability of the library’s digital infrastructure. The empathy dimension is represented by understanding and support in digital reading. Specifically, this involves the library’s ability to empathize with users’ requirements, its ability to provide personalized assistance, and its ability to guide responses to individual digital reading preferences and challenges. By mapping these dimensions to a service quality model, we created a comprehensive framework for assessing how different dimensions of digital reading services affect reader service satisfaction in college libraries. Based on this theoretical framework, we could explore the interactions between these digital reading service dimensions and their impact on the service quality in college libraries, and two propositions are proposed.

Proposition 1. *Solitary digital reading service dimensions*, *such as access and usability*, *resource consistency*, *timely assistance*, *credible services*, *and user support*, *cannot lead to improvements in reader service satisfaction in college libraries*.

Proposition 2. *Appropriate configurations of digital reading service dimensions lead to a high level of reader service satisfaction in college libraries*.

Digital reading services in college libraries are inherently multifaceted. Libraries have to address the different needs, preferences, and expectations of different groups of students and provide a diversified range of services. Different digital reading services play crucial roles in college libraries, including providing digital access to resources, ensuring availability of resources, maintaining consistency of resources, providing just-in-time assistance, ensuring trustworthy services, and providing user support. However, taking these dimensions on an isolated basis may ignore the complexity of reader service satisfaction of digital reading in college libraries. The interdependence of these dimensions needs a more holistic approach to understanding and improving the digital reading service in college libraries.

Previous studies demonstrate that reader service satisfaction is a holistic measure that is affected by different dimensions of library services [19]. In college libraries, student satisfaction depends on more than simply the accessibility of digital resources or the responsiveness of library staff. It would be a combination of multiple factors, such as the availability of digital platforms, reliability and consistency of digital resources, availability of timely assistance, trustworthiness of services, and empathetic user support. For example, whether students can easily access digital resources is frequently related to the usefulness of the digital platform itself. If the platform is difficult to navigate or has complex search functions, students cannot locate and utilize resources effectively. As well, the consistency of resources has a direct impact on reliability. If resources are frequently inaccessible or have technical problems, this weakens the reliability of library services [21].

Measuring reader service satisfaction in college libraries is not an aggregation of reader service dimensions. Instead, it is the result of the interaction and coordination of these dimensions. For instance, just improved service credibility may not significantly affect satisfaction if the usability of digital reading, resource consistency, timely help, and user support are not present. This study recognizes the intricate relationship between different digital reading dimensions and reader service satisfaction and emphasizes the importance of understanding how these collectively contribute to overall reader service satisfaction. By revealing the interdependence of these dimensions, college libraries can better customize their services to meet the different needs of readers and thus enhance readers’ service satisfaction. Therefore, Proposition 1 proposes the interrelatedness of different service dimensions in shaping reader service satisfaction in college libraries.

It is critical to consider the multiple dimensions of college library services and the inherent complexity of reader service satisfaction. Existing research suggests that improving reader service satisfaction is not a single task, but involves multifaceted considerations to meet the different needs and expectations of library readers to enhance reader service satisfaction [7]. Some studies of readers’ service satisfaction measurement emphasized the interconnections between various service dimensions and pointed out that different service dimensions need to be configured to meet different readers’ needs, thereby enhancing the overall quality of library services [23]. The correlations between the different dimensions of reader services revealed by existing studies underscore the importance of building and harmonizing the configuration of these dimensions to enhance services quality.

Research on the service quality model has also emphasized the importance of considering how different service dimensions interact with each other to influence customer perceptions [24]. The service quality model takes into account the interconnections and synergies between different service dimensions. In the same way, reader service satisfaction in the college library context is not the result of an overlay of each dimension. On the contrary, reader service satisfaction is measured by the dynamic interactions between different reader service dimensions. This is consistent with the larger literature on service quality, which suggests that good reader service often depends on the harmonization and coordination of different aspects of the service [25].

To achieve high levels of satisfaction with reader services in college libraries, the attention should shift from isolated improvements in a single reader service dimension to identifying and establishing appropriate configurations. Moreover, these configurations should be regarded as interrelated and complementary, and they should jointly create a positive and satisfying service experience for library readers. By considering specific combinations that can lead to high levels of reader service satisfaction, this study aims to provide practical insights and guidance for college libraries to improve their digital reading service quality. Thus, we believe that the way for college libraries to achieve high levels of reader service satisfaction lies in identifying and establishing appropriate configurations of the different digital reading service dimensions

## 4. Research methodology

### 4.1 Data

This study was conducted over a six-month period to collect data with the aim of gaining a comprehensive understanding of the elements influencing reader service satisfaction in college libraries. The data collection period began in March 2023 and ended in August 2023. This period allowed us to capture potential changes in reader service satisfaction across different periods. To measure the factors that affect reader service satisfaction in college libraries, a structured questionnaire was used. The questionnaire was designed to capture responses from students who regularly use college library services, particularly digital reading services, and the participants in our study all come from a single college. The study was conducted at a Chinese college library, an academic institution known for its commitment to digital transformation initiatives in its library services. The library serves as a central resource hub for students, faculty, and researchers. With an extensive collection of approximately 2,500,000 books, journals, and multimedia resources, including special collections such as archives of rare manuscripts and historical documents, the library caters to a diverse academic community. Users span various demographics, including undergraduate and postgraduate students, as well as faculty from different departments, contributing to the rich tapestry of our research environment. During the data collection period, 428 questionnaires were collected. The questionnaires were distributed both within the library and through an online platform, ensuring a broad and diverse sample of library users.

Specifically, the survey was administered in the library setting to capture responses from patrons who were physically present within the library premises. We chose this approach to ensure that we could directly engage with library users and gather feedback from those who regularly utilize library services, including digital reading resources. The survey was made available in print format for those who stayed in the library. We ensured that the survey distribution process in the library was conducted during times of regular library activity to maximize participation. In addition to conducting the survey in the library, we also distributed it online to reach a broader audience beyond those who visit the physical library. The online distribution method allowed us to collect responses from individuals who may not have immediate access to the library but still engage in digital reading activities. The survey link was shared through various channels, including college email lists and social media platforms, inviting individuals to participate in the survey and share their experiences with digital reading services in the college library. We employed a rigorous research methodology by opting for random sampling in the administration of the questionnaire survey. This approach involved selecting participants randomly from the population of library users, ensuring that each member had an equal chance of being included in the study. By utilizing random sampling, we aimed to enhance the generalizability of our findings to the broader library user community, thereby strengthening the validity and reliability of our research outcomes. Participants were provided written informed consent that the data collected would be used only for academic research, and their personal information would not be disclosed. Table 1 shows the participants’ profiles.

**Table 1:** Participants profiles (*N* = 396).

Measures	Items	Frequency	Percent
Gender	Male	171	43.18
	Female	225	56.82
Age	Under 20	59	14.90
	21−25	288	72.73
	Over 25	49	12.37
Education	Bachelor’s	285	71.97
	Master’s (above)	111	28.03
Household size	1–2	62	15.65
	3–4	238	60.10
	5–6	79	19.95
	Above 6	17	4.29
Experience in using digital reading services	1−3 years	313	79.04
	3−5 years	15	3.78
	Over 5 years	68	17.17

To ensure questionnaire quality and reliability, we designed it based on existing research [26], emphasizing validity and relevance. Clear guidelines, respondent anonymity, and an emphasis on feedback importance aimed to elicit honest responses. Rigorous data analysis, including statistical tests and validation procedures, identified and addressed inconsistencies. Questionnaires with incomplete responses (over 6) were considered invalid and were subsequently deleted from the dataset. Moreover, questionnaires that exhibited patterns of duplicate responses (all questions had the same options) were identified as invalid and removed from the dataset. We utilized 396 valid questionnaires for result analysis, maintaining a focus on our core research objectives—providing reliable insights into factors influencing reader service satisfaction in college libraries.

### 4.2 Measures

To assess the various dimensions of digital reading services, including access and usability, resource consistency, timely assistance, credible services, and user support, we utilized a seven-point Likert scale [27]. This scale is a widely accepted and validated method for measuring perceptions and attitudes in research. Respondents were asked to rate their agreement with specific statements regarding each dimension, with response options ranging from "1—strongly disagree or significantly lower" to "7—strongly agree or significantly higher" [28]. For all measurement items, methods from previous literature were used to measure the above dimensions of digital reading services. The six variables and their corresponding measurement items are shown in Table 2.

**Table 2:** Measurement items.

Variable	Code	Items	References
Access and usability	AU1	The digital reading platform is easy to navigate.	Nielsen and Levy (1994)
	AU2	I can quickly find the digital resources I need.	
	AU3	Digital reading materials are accessible from various devices.	
Resource consistency	RC1	The digital resources are consistently available.	Uzir et al. (2021)
	RC2	I rarely encounter technical issues when accessing digital materials.	
	RC3	The library’s digital collection is regularly updated and expanded.	
Timely assistance	TA1	Library staff responded promptly to my queries related to digital reading.	Venkatesh et al. (2017)
	TA2	I can easily find assistance when I encounter difficulties with digital reading.	
	TA3	The library provides adequate support for troubleshooting digital reading problems.	
Credible services	CS1	I trust the accuracy and authenticity of the digital resources provided by the library.	German et al. (2022)
	CS2	The digital services offered by the library are reliable and secure.	
	CS3	I have confidence in the library’s digital services.	
User support	US1	The library provides user-friendly guides and tutorials for digital reading.	Uzir et al. (2021)
	US2	I receive helpful guidance when seeking assistance from library staff.	
	US3	The library offers personalized support to meet my specific digital reading needs.	
Reader service satisfaction	RSS1	I intend to use the digital reading service in college libraries.	Pappas et al. (2020)
	RSS2	I intend to encourage others to use the digital reading service in college libraries.	
	RSS3	I predict that our society will predominantly support the use of digital reading services in college libraries.	

Specifically, the dimension of access and usability aimed to capture respondents’ perceptions of the convenience of accessing digital reading materials and the usability of digital reading platforms. We used the scale developed by Nielsen and Levy [29] to measure this dimension. To assess the consistency of digital resources, the following items proposed by Uzir et al. [30] were employed. For timely assistance, this dimension assessed the availability and efficiency of assistance when users encounter issues. Following Venkatesh et al. [31], we used three items to measure this dimension. To measure the credibility of digital services, the measurement items proposed by German et al. [32] were included. To assess the user support dimension, we utilized a scale developed by Uzir et al. [30]. This dimension aimed to assess the level of support and guidance offered to users in digital reading.

In the construction of our research dimensions and variable items, it is crucial to explicitly acknowledge that the selected measurement items, as outlined in Table 2, were primarily drawn from usability testing studies. Additionally, the selected scale items, encompassing access and usability, resource consistency, timely assistance, credible services, and user support, hold particular significance within the context of our study on digital transformation in college libraries. Each of these dimensions plays a pivotal role in shaping the user experience and satisfaction with digital reading services. Access and usability address the ease with which users can navigate and interact with digital resources, directly impacting the overall service satisfaction. Resource consistency ensures a stable and reliable digital environment, contributing to a seamless reading experience. Timely assistance becomes critical in addressing user queries or issues promptly, fostering a positive perception of the library’s services. Credible services reflect the trustworthiness and authenticity of digital resources, a paramount concern in academic settings. Finally, user support is instrumental in providing guidance and assistance throughout the digital reading journey. Together, these dimensions form a comprehensive framework for evaluating and enhancing digital reading services in college libraries, aligning with the evolving needs of users in the digital age.

### 4.3 Validity and reliability

The validity and reliability of the scales were examined in this study. First, the validity of the scale was verified by assessing the items loadings of all variables. Specifically, we used confirmatory factor analysis (CFA) to check whether the items loaded strongly on the structures [33]. The loadings indicate the strength and importance of the relationship between each item and its underlying variables [34]. Table 3 shows the loadings for each item, where the loadings for each item ranged from 0.607 to 0.863 (greater than 0.6), indicating that the scales used in this study were valid [35].

**Table 3:** Assessment of reliability and convergent validity.

Variables	Items	Loading	CR	AVE
Access and usability	AU1	0.854	0.868	0.564
	AU2	0.654		
	AU3	0.732		
Resource consistency	RC1	0.620	0.783	0.550
	RC2	0.744		
	RC3	0.644		
Timely assistance	TA1	0.691	0.776	0.537
	TA2	0.762		
	TA3	0.743		
Credible services	CS1	0.607	0.782	0.549
	CS2	0.863		
	CS3	0.731		
User support	US1	0.756	0.828	0.616
	US2	0.740		
	US3	0.854		
Reader service satisfaction	RSS1	0.801	0.812	0.592
	RSS2	0.813		
	RSS3	0.688		

Second, we employed a reliability test to assess the internal consistency of the scales used in the study. We calculated the composite reliability (CR) for each variable. Composite reliability measures the degree of correlation between the items in a variable and the joint contribution of these items to measure the intended variable [36]. A high CR value indicates that the items in the scale provide a reliable measure of the variable. In the current research, a CR value higher than 0.7 is considered acceptable, which indicates that the scale is reliable [37]. The results of the reliability test (Table 3) showed that the CR of all variables is above 0.7, and thus the scales used in this study were reliable [38].

Third, we utilized the Average Variance Extracted (AVE) method [39] to evaluate the discriminant validity of the variables. Discriminant validity assesses whether a variable is distinct from others, ensuring that the measures are not too closely related [40]. We compared the AVE of each construct to the squared correlations between that construct and other constructs. The results of discriminant validity (Table 4) indicated that the AVE values of all variables were higher than the squared inter-construct correlations. Therefore, the variables had good discriminant validity. By assessing the loadings of each item, internal consistency using CR, and discriminant validity via AVE, we ensured that the scales used in this study were both valid and reliable. These analyses ensured that the scales accurately captured the intended dimensions and were consistent in assessing satisfaction with digital reading services and reader services in college libraries.

**Table 4:** Discriminant validity.

Variables	AVE	1	2	3	4	5	6
1. Access and usability	0.564	0.719					
2. Resource consistency	0.550	0.563	0.632				
3. Timely assistance	0.537	0.588	0.458	0.698			
4. Credible services	0.549	0.199	0.351	0.526	0.764		
5. User support	0.616	0.504	0.473	0.489	0.243	0.633	
6. Reader service satisfaction	0.592	0.393	0.324	0.495	0.444	0.532	0.723

### 4.4 Method

This study used a mixed-method approach, integrating Qualitative Comparative Analysis (QCA) [41] and Fuzzy Set Qualitative Comparative Analysis (fsQCA) [42, 43] to explore the multifaceted influence of digital reading on reader service satisfaction in college libraries. Qualitative comparative analysis is a methodology used to examine complex causal relationships and identify specific configurations that lead to specific results. The fsQCA method extends the QCA methodology by providing for the integration of fuzzy sets and recognizing the gradual membership of cases in different sets [44]. In this regard, the fsQCA method could resolve the cases that cannot be categorized into the binary category.

Different from traditional regression analysis methods that assume the existence of linear relationships and require a large number of samples, the fsQCA method is well suited to study complex nonlinear relationships with smaller sample sizes, which is often used in qualitative research [45, 46]. In this study, we aim to understand the different dimensions of digital reading and their interactions in influencing reader service satisfaction. Traditional regression models often struggle to capture the intricate interdependencies between the multiple dimensions of reader service, while the fsQCA approach could identify specific combinations of conditions that lead to the result [47].

In addition, the fsQCA method is suitable for analyzing multifaceted nonlinear relationships and can be adapted to the complexity of readers’ service satisfaction in college libraries [48, 49]. Previous research employed the fsQCA method, showcasing its applicability in analyzing complex and nonlinear relationships, and they demonstrated the method’s effectiveness in handling intricate relationships. The choice of fsQCA is informed by the method’s demonstrated utility in studies similar to this study, despite a different thematic emphasis. This approach allows us to leverage the methodological strengths of previous research, adapting them to our specific investigation of reader service satisfaction within the unique setting of college libraries. In reader service satisfaction studies, user experiences can vary widely, and the fsQCA methodology allows for the consideration of gradual membership, ensuring a more accurate representation of the complexity of reader services. As the main methodology of this empirical study, the fsQCA method is an appropriate choice for exploring the interaction of digital reading dimensions in affecting reader service satisfaction. Therefore, the fsQCA method is used to explore the complex relationships between the dimensions of digital reading services and their impact on reader service satisfaction.

The fsQCA model consists of several key components: fuzzy sets, identification of conditions and outcomes, construction of truth tables, assessment of sufficiency and necessity, and sensitivity analysis. Fuzzy sets involve representing variables with degrees of membership rather than strict categorization, enabling a nuanced understanding of relationships. The identification of conditions and outcomes involves selecting relevant factors believed to influence the outcome of interest, such as demographic characteristics or organizational practices. Through the construction of truth tables, researchers systematically evaluate all possible combinations of conditions and their associated outcomes. The assessment of sufficiency and necessity utilizes Boolean minimization techniques to determine which combinations of conditions are both sufficient and necessary for producing the outcome. Finally, sensitivity analysis ensures the robustness of the findings by examining the impact of variations in membership scores and calibration thresholds on the results.

## 5. Results

### 5.1 Calibration

To analyze the collected survey data, we calibrated mean scores to fuzzy set membership values, a crucial step for fuzzy set qualitative comparative analysis (fsQCA). First, we converted respondents’ 7-point Likert scale ratings into fuzzy membership values ranging from 0 to 1 [50]. Second, we established qualitative thresholds to denote full membership and full non-membership in the fuzzy set. Full membership was set at a fuzzy membership score of 1, indicating complete agreement, while full non-membership was set at 0, indicating complete disagreement [28]. To establish a continuum between full members and full non-members, we set the intersection point at a Likert scale rating of 4. This point signifies a neutral attitude where respondents neither strongly agree nor strongly disagree. These defined thresholds and intersection points facilitated the conversion of discrete Likert scale responses into fuzzy set membership values. Consequently, the fsQCA method enables exploration of the multifaceted effects of digital reading on reader service satisfaction in college libraries.

### 5.2 Analysis of necessary conditions

In the analysis of necessary conditions, we explored whether any single condition within the dimensions of digital reading services was necessary for high reader service satisfaction in college libraries. In the fsQCA model, a necessary condition must be present for the outcome to occur [9, 46]. The results of the necessary conditions analysis (Table 5) showed that none of the conditions were necessary to achieve high reader service satisfaction. This finding underscores the complexity of the relationships between digital reading services and reader service satisfaction in college libraries. It suggests that no single dimension, such as access and usability, resource consistency, timely assistance, credible services, or user support, is sufficient to guarantee high reader service satisfaction.

**Table 5:** Necessity of the conditions relative to the occurrence and no occurrence of reader service satisfaction in college libraries.

Condition	Reader service satisfaction		~ Reader service satisfaction	
	Consistency	Coverage	Consistency	Coverage
Access and usability	0.321	0.689	0.532	0.609
~ Access and usability	0.680	0.818	0.709	0.354
Resource consistency	0.641	0.836	0.831	0.444
~ Resource consistency	0.574	0.754	0.694	0.442
Timely assistance	0.499	0.795	0.688	0.506
~ Timely assistance	0.725	0.849	0.858	0.412
Credible services	0.353	0.794	0.580	0.601
~ Credible services	0.842	0.830	0.797	0.362
User support	0.606	0.787	0.805	0.482
~ User support	0.646	0.790	0.812	0.458

The necessity for multiple dimensions stems from the inherent complexity and multifaceted nature of user expectations and experiences in the digital realm. By employing a rigorous analysis of user feedback, preferences, and service usage patterns, we discerned that a singular dimension fails to encapsulate the diverse aspects influencing reader service satisfaction. Our study goes beyond the conventional unidimensional assessments and recognizes that the satisfaction of users engaging with digital reading services is contingent upon various interrelated factors. Through advanced statistical techniques and data-driven methodologies, we identified distinct dimensions, such as access and usability, resource consistency, timely assistance, credible services, and user support, each playing a pivotal role in shaping user satisfaction. The correlation and interdependence among these dimensions were established through the application of the fsQCA approach, allowing us to uncover specific combinations that contribute synergistically to heightened levels of reader service satisfaction. Furthermore, our analysis revealed that a singular focus on any isolated dimension might not capture the holistic satisfaction landscape. Users perceive satisfaction as a collective outcome of seamless access, reliable resources, prompt assistance, credibility, and effective support. This understanding guided our decision to adopt a multi-dimensional framework, ensuring a more comprehensive and nuanced evaluation of the evolving role of college libraries in the digital age.

The lack of necessary conditions underscores the interactions between the multiple dimensions of digital reading services to increase reader service satisfaction. This is consistent with the theoretical framework and proposition 1, which emphasizes the interdependence of these dimensions and the specific configurations to achieve high reader service satisfaction. Based on the results of the necessary conditions analysis, we conducted a further conditional configuration analysis to identify the specific combinations of conditions in the digital reading service dimensions that can lead to high reader service satisfaction.

### 5.3 Analysis of sufficient conditions

After fuzzy set correction and necessary condition analysis, we established a truth table to examine the distribution of sample cases for all possible combinations of causal conditions within the dimension of digital reading services in college libraries as well as their effects on reader service satisfaction. The truth table includes two key criteria. First, the frequency values represent the number of cases in the dataset that exhibit each particular combination of conditions [48]. This criterion allows us to determine which configurations are more prevalent among respondents. Second, the consistency threshold is used to determine the minimum level of consistency required for a configuration to be considered empirically relevant [51]. This criterion ensured that the identified configurations were sufficiently consistent and not just randomly occurring.

We identified three solutions based on the Quine-McCluskey algorithm [9]: a complex solution, a parsimonious solution, and an intermediate solution. The complex solution included all configurations, regardless of their consistency or frequency. On the other hand, the parsimonious solution only retained the most consistent configurations but might ignore some configurations with lower frequencies. The intermediate solution balanced both frequency and consistency. We chose the intermediate solution to explain the final configurations because this solution ensures that the identified configurations are not only empirically consistent but also have a reasonable frequency.

The intermediate solution provides a robust foundation for interpreting the multifaceted impact of digital reading on reader service satisfaction in college libraries. By selecting the intermediate solution, we aimed to strike a balance between relevance and comprehensiveness, enabling us to draw meaningful insights while avoiding the exclusion of potentially valuable configurations that might be omitted in the parsimonious solution. The interpretation of the final configuration by choosing an intermediate scenario is consistent with the goals of this study, which are to capture the complexity of digital reading service and to understand the combinations of conditions that contribute to high reader service satisfaction in college libraries.

Table 6 shows the results of the intermediate solution for high reader service satisfaction in college libraries. The results showed that the overall solution consistency was 0.894, and the overall solution coverage was 0.703. The high overall solution consistency indicates that the identified configurations, comprising combinations of digital reading dimensions, are highly internally consistent. This means that the specific conditions and interplay among the dimensions within each configuration consistently lead to high levels of reader service satisfaction. The overall solution coverage of 0.703 indicates the extent to which the identified configurations account for high reader service satisfaction. In other words, these configurations explain approximately 70.3% of the instances of high reader service satisfaction in college libraries.

**Table 6:** Results from fsQCA.

Configuration	1	2	3	4	5	6	7
Access and usability	•	⊗	⊗		⊗	⊗	
Resource consistency			•	●	•		⊗
Timely assistance	⊗		•			•	●
Credible services	⊗	⊗		⊗			⊗
User support		●		•	●	●	⊗
Consistency	0.914	0.947	0.927	0.945	0.936	0.953	0.975
Raw Coverage	0.469	0.498	0.381	0.434	0.439	0.382	0.282
Unique Coverage	0.071	0.066	0.012	0.015	0.007	0.008	0.015
Overall solution consistency	0.894						
Overall solution coverage	0.703						

Note: Black circles(●) indicate the presence of a condition, and circle with “x”(⊗) indicate its absence. Large circle: core condition, Small circle: peripheral condition, Blank space: “do not care” condition.

In Solution 1, we observed that readers with high service satisfaction considered the library’s digital resources to be easily accessible and usable, which emphasizes the importance of access and usability in the digital reading experience. However, this configuration was accompanied by negativity towards timely assistance and credible service. This configuration implies that ease of access and availability contribute significantly to satisfaction, and readers may be willing to overlook the lack of timely help or concerns about the credibility of the service. The interactions between different reader service dimensions in this solution suggest that a well-designed, user-friendly digital reading environment can bridge potential weaknesses in the library’s ability to provide timely assistance or concerns about the credibility of the library’s services. It also emphasizes the importance of ensuring accessibility and usability of digital resources to increase reader satisfaction.

Solution 2 presents a unique contrast to Solution 1. In this configuration, the core condition for achieving high reader service satisfaction is the negation of access and usability, which reveals that readers may still have a high level of satisfaction even if they think the digital resources are less accessible or usable. However, a key factor in this solution is the presence of user support. This suggests that strong user support services play a compensatory role in enhancing reader satisfaction. The peripheral condition for Solution 2 is the credible service, which suggests that the credibility of the service remains a key factor regardless of access and availability concerns. This solution underscores the trade-off between accessible service and user support. Moreover, it implies that user support can also be a valuable palliative factor while access and availability of reader service are important.

Solution 3 offers a distinct perspective on reader service satisfaction. The peripheral condition for high satisfaction in this configuration comprises the negation of access and usability, resource consistency, and timely assistance. This suggests that readers can achieve high satisfaction even if they find low accessibility and usability of digital resources, encounter inconsistency of resources, and lack of timely help. The presence of resource consistency and timely assistance may be less critical in this context. The consistency of resources and timely assistance may not be as important in this situation. It suggests that while resource consistency and timely assistance are valuable dimensions, readers may prioritize other dimensions of the digital reading experience, such as the content or its user-friendliness.

Solution 4 identifies resource consistency as a core condition for high reader service satisfaction. However, this condition was paired with the negative condition of credible service, which suggests that readers may be highly satisfied if they perceive that the library’s digital resources are consistently available and reliable, even if they have doubts about the credibility of the service. The peripheral condition includes user support, suggesting that the availability of support services further increases reader satisfaction. This configuration highlights the dynamic relationship between resource consistency, credibility service, and user support in affecting reader service satisfaction. In this solution, readers seem to prioritize the reliability and consistency of digital resources, which suggests that the availability of a strong collection is an important determinant of reader satisfaction. Although credible services played a role, user support was also considered a valuable dimension of this solution.

Solution 5 focuses on the negation of access and usability as the core condition and shows that readers can achieve high levels of satisfaction even if they perceive poor accessibility and usability of digital resources. The peripheral condition is the negation of resource consistency, which emphasizes the trade-off between resource consistency and user support in this configuration. In this solution, user support is prioritized over resource consistency. Readers are likely to accept the lack of consistent resources if they have access to strong user support. Solution 5 emphasizes the dynamic nature of digital reading and the role of user support in bridging the lack of resource consistency. In addition, this configuration shows the various factors that influence reader service satisfaction and the trade-offs that readers may make.

Similar to Solution 5, Solution 6 presents the negation of access and usability as the core condition and reveals that readers can achieve high satisfaction even if they perceive poor accessibility and usability of digital resources. This core condition is also accompanied by the presence of user support. However, in this configuration, the peripheral condition includes a negation of timely assistance, suggesting that readers may not consider timely assistance to be as important in this situation. This solution emphasizes the importance of access and usability of digital resources as well as the availability of user support in affecting reader service satisfaction. In this solution, readers prioritize the digital reading experience and the availability of support rather than timely assistance.

In Solution 7, timely assistance emerges as the core condition for high reader service satisfaction, accompanied by the negation of user support. This suggests that readers can achieve high satisfaction if they receive timely assistance even if user support is lacking. The peripheral condition includes the negation of resource consistency and the negation of credible services, which indicate that resource consistency and credible services may be less critical in this condition. This configuration highlights the significance of timely assistance in the digital reading experience and how it can compensate for the lack of user support. It also suggests that resource consistency and credibility might be secondary considerations for readers who prioritize timely assistance.

These seven solutions highlight the multifaceted nature of reader service satisfaction in college libraries. They illustrate those different configurations of digital reading service dimensions can lead to high satisfaction. The interplay between access and usability, resource consistency, timely assistance, credible services, and user support varies across these configurations, emphasizing the complexity and diversity of factors influencing service quality in college libraries. Each solution provides valuable insights into the configurations that contribute to high reader service satisfaction.

In summary, the fsQCA results emphasize the delicate balance of various aspects of digital reading services and their different impacts on reader service satisfaction in college libraries. Two propositions proposed by this research can also be verified. First, the fsQCA results indicate that solitary digital reading service dimensions cannot lead to improvements in reader service satisfaction in college libraries (Proposition 1). Second, the results indicate that appropriate configurations of digital reading service dimensions can lead to a high level of reader service satisfaction in college libraries (Proposition 2).

Considering the potential divergence in preferences and satisfaction factors among readers with limited experience in using digital reading services, we conducted an analysis on individuals with experience in using digital reading services of less than 1 year. This could allow us to unravel distinctive patterns and configurations that might differ from the broader survey results on reader service satisfaction. We also collected a robust dataset consisting of 167 questionnaires from readers lacking experience in digital reading services. This dataset, consisting of 167 questionnaires, was collected through a separate survey specifically targeting novice users. It is important to note that this dataset is independent of the original 428 collected questionnaires. Employing the same meticulous procedures outlined earlier, we applied the fsQCA model to this dataset. The subsequent analysis, incorporating fuzzy set correction and necessary condition assessments, produced findings on the factors influencing reader service satisfaction among individuals with limited exposure to digital reading services. For readers lacking experience with digital reading services, our findings reveal distinctive patterns that deviate from the overall trends in reader service satisfaction.

Notably, solutions 3, 6, and 7 present unique patterns, while the remaining configurations converge in their outcomes. Specifically, in Solution 3, we observed a discrepancy with the overall findings. For readers lacking experience with digital reading services, resource consistency and timely assistance emerge as crucial dimensions for satisfaction, while access and usability become less pivotal. This configuration implies that, for this specific group, the reliability of resources and timely support play more central roles in shaping satisfaction compared to other aspects of the digital reading experience. Contrary to Solution 6 in the overall findings, readers lacking experience with digital reading services exhibit variations from their counterparts in the overall findings. These configurations underscore the nuanced balance between access and usability, user support, and timely assistance for readers lacking experience with digital reading services. Timely assistance, although less critical in the overall findings, emerges as a more influential factor for this subgroup, demonstrating the distinct considerations of digital novices. Solution 7 showcases a shift in priorities for readers lacking experience with digital reading services. While timely assistance remains a core condition, the absence of user support is more tolerable for this subgroup. This suggests that timely assistance plays a pivotal role in satisfying digital novices, potentially compensating for the lack of user support, resource consistency, and credible services.

These nuanced configurations highlight the specific needs and preferences of readers lacking experience with digital reading services. The distinct patterns identified in this subgroup analysis provide valuable insights into tailoring digital reading services to cater to the unique expectations of digital novices, contributing to a more comprehensive understanding of reader service satisfaction in college libraries. While there are subtle variations in some solutions, the overarching trends align with the overall findings. Results all underscore the complexity of factors influencing satisfaction, emphasizing the need for a holistic approach to digital reading services. Furthermore, the resonance of these configurations with the overall results reinforces the second proposition. The consistent observation is that appropriate combinations of digital reading service dimensions are pivotal in achieving high levels of reader service satisfaction in college libraries.

## 6. Discussions

### 6.1 Research findings

This research explored the multifaceted influence of different digital reading dimensions on reader service satisfaction in college libraries. Based on the service quality model, we employed the fsQCA method to verify the intricate relationships between various dimensions of digital reading services. We revealed the different configurations of conditions that lead to high reader service satisfaction. With the digital era continuing to shape college libraries, it is critical to understand the specific conditions that lead to high reader satisfaction.

One of the key findings of this study is the existence of multiple configurations of digital reading dimensions that contribute to high reader service satisfaction. These configurations revealed through the fsQCA analysis, underscore the complexity of service quality in college libraries. This study identifies solutions where factors like access and usability, resource consistency, timely assistance, credible services, and user support interact in diverse ways to influence reader satisfaction. This finding is consistent with previous research that emphasized the multiple dimensions of library service quality [52]. However, the unique aspect is the deep understanding of these configurations and the interactions between the dimensions. Some configurations emphasize the importance of convenient user access, while others prioritize resource consistency or timely assistance. This finding advances the understanding of the complex and changing service within college libraries, revealing unique patterns that could develop reader service.

Our analysis identified some configurations where compensation effects played an important role in achieving high reader service satisfaction. For example, we observed that accessibility and usability compensated for the lack of timely assistance and service credibility (Solution 1). Conversely, user support compensated for the lack of accessibility and usability (Solution 2). These findings emphasize that readers may prioritize some aspects and tolerate deficiencies in others. This observation coincides with previous research on service quality, which has explored how users weigh certain aspects through the presence of compensating factors [5]. Our findings reveal these tradeoffs in digital reading and guide libraries to improve their services. These compensatory effects provide strategies for optimizing limited resources and tailoring services to meet different reader requirements.

Third, we uncovered the dynamic nature of patron service satisfaction in college libraries and the role of user preferences. Solution 3 reveals that readers may prioritize other aspects besides resource consistency and timely assistance, which suggests the flexibility of user preferences. Therefore, libraries should focus on addressing the various needs and preferences of readers to increase their satisfaction. While previous studies have emphasized the importance of developing user-centered services [53], the findings of this study provide specific insights into the aspects that are most important to readers in different situations. Understanding these changes in reader preferences could help libraries provide more tailored and responsive services.

### 6.2 Theoretical implications

This study has important theoretical significance on how to improve college library service quality in the digital era. The library service quality has been assessed through different dimensions, and previous studies usually examined these dimensions in isolation [54]. However, this study shows that reader service satisfaction is the result of a specific configuration and interaction between these dimensions rather than a simple summation of individual dimensions. This calls for a reconceptualization of service quality in college libraries, moving from a single-dimension approach to a more holistic understanding that recognizes the complex relationships and trade-offs between different service dimensions. Thus, this study could not only contribute to the theoretical foundation of library science but also provide a framework for capturing the changing dynamics of service quality in the digital era.

This study also emphasizes the value of using fuzzy set qualitative comparative analysis as a research method in library science. The fsQCA method allows for the study of complex nonlinear relationships between variables, and thus it is particularly appropriate for examining the multifaceted influences on reader service satisfaction. The application of fsQCA in reader service satisfaction provides a new perspective on service quality research. The fsQCA method also demonstrated that it could reveal complex structures and patterns that may be overlooked by traditional quantitative approaches. By extending the methods available to library science researchers, this study contributes to a broader discussion of innovative research methods and their applicability to understanding and enhancing library services.

The theoretical implications of this study also include the revelation of the importance of user-centeredness and service adaptability in college libraries. The different configurations of digital reading dimensions identified by the findings of this study emphasize the dynamic nature of reader preferences. Libraries need to recognize that users’ needs and expectations are not uniform, and thus they should strive for flexibility in designing their services. This is consistent with previous research which revealed the important role of libraries in meeting the diverse and changing needs of their readers [55]. Therefore, libraries should adopt a more adaptive and user-centered approach to improve service quality. In this way, libraries will be better able to adapt to the different preferences and improve the overall service quality.

### 6.3 Practical implications

Our study reveals important insights into how different digital reading interfaces impact user satisfaction, offering practical implications primarily for vendors and secondarily for academic librarians. One of the practical implications of this study is that vendors need to customize their digital reading services to different preferences. The findings of the study emphasize that there is no one-size-fits-all solution for improving reader service satisfaction. Instead, vendors should invest in customizing their services according to their specific user groups and needs. For example, Solution 3 suggests that consistency of resources and timely assistance may not be as important to some readers, allowing vendors to allocate resources more strategically. By conducting user surveys, analyzing usage data, and actively seeking feedback, vendors can gain insights into the specific preferences and priorities of their users to inform the design and delivery of digital reading services. This practical approach helps to create a more user-centered and adaptable digital environment.

Second, the results of this study highlight the importance of user support services in increasing reader service satisfaction. Vendors should recognize that knowledgeable and responsive support staff can serve as a valuable compensating factor, especially when certain aspects of digital reading may be inadequate. Our findings underscore the integral nature of well-structured and proficient user support services in fostering positive reader service satisfaction. Vendors that prioritize training and cultivating knowledgeable staff significantly contribute to an enhanced user experience. User support services act as a linchpin in the digital reading landscape, ensuring that users receive the assistance they need, thereby reinforcing overall satisfaction. This practical implication emphasizes the need for vendors to invest in the training and development of support staff to ensure that they are capable of providing effective assistance to readers [56]. In addition, online assistance resources, guides, and tutorials can serve as important tools for providing user support. By prioritizing user support, vendors can create a more supportive and inclusive environment for readers, and thus improve their service quality.

Third, this paper underscores the importance of accessibility and usability of digital resources in improving reader service satisfaction. Our study elucidates how effective management of these elements directly contributes to heightened levels of reader service satisfaction. Vendors that prioritize intuitive design, user-friendly interfaces, and seamless access to digital resources create an environment where users can navigate, retrieve, and engage with content effortlessly. Our findings provide clear guidance for vendors looking to improve their services in the context of digital transformation. Recognizing the critical importance of accessibility and usability empowers vendors to tailor their digital reading services to meet the evolving needs and preferences of users effectively. Incorporating strategies to enhance the accessibility and usability of digital resources not only enhances user satisfaction but also fosters a more inclusive and accommodating digital environment. Vendors should prioritize ensuring that their digital platforms are easily accessible and user-friendly. This practical implication requires the development of intuitive, accessible digital platforms that are easy to navigate. Vendors should also consider providing training and workshops to help users browse digital resources effectively. By improving accessibility and usability, vendors can create a more satisfying digital reading experience for their users.

While the primary responsibility for interface improvements lies with vendors, librarians also play a crucial supportive role. Librarians can provide valuable training and advice to users, helping them navigate and utilize available digital reading tools more effectively. This support is essential, especially as a temporary measure, until vendors implement necessary improvements. Librarians should focus on teaching users how to maximize the benefits of well-designed tools rather than compensating for poorly designed products. Apart from the internal efforts of libraries, the collaboration between libraries and vendors plays a pivotal role in enhancing digital reading services and ultimately improving user satisfaction. Vendors of digital reading platforms are essential partners in the delivery of satisfactory user experiences within college libraries. First, vendors should prioritize user feedback and actively solicit input from library professionals and patrons to identify areas for improvement. This collaborative approach ensures that digital reading platforms are designed and updated in a manner that aligns with the specific requirements and preferences of library users. Second, vendors play a crucial role in providing ongoing support and technical assistance to libraries, ensuring seamless integration and optimal functionality of digital reading platforms. Moreover, vendors are encouraged to invest in research and development initiatives aimed at enhancing the features and capabilities of their products. By staying abreast of emerging trends and technological innovations, vendors can proactively address potential issues and deliver innovative solutions that enhance the overall user experience.

### 6.4 Limitations and suggestions for future research

While this study provides valuable insights into reader service satisfaction in the context of college libraries, it also has inherent limitations. The limitation of this study is the specificity of its context. Our research unveiled the existence of multiple configurations of digital reading dimensions contributing to high reader service satisfaction, emphasizing the intricate relationships between various dimensions. While our study context was specific to one institution, the broader implication is the universality of these configurations across college libraries. Recognizing this, future research should conduct similar studies in diverse environments to explore generalizability. The identified configurations, highlighted in our key findings, serve as a foundational framework that can be applied and adapted across various institutions, user demographics, and cultural contexts [57].

A second limitation is the dynamic nature of user preferences and evolving technology. Our findings illuminated the dynamic nature of patron service satisfaction in college libraries, emphasizing the flexibility of user preferences. This aligns with the dynamic nature of digital reading services and evolving user expectations. As we suggest future research to conduct longitudinal studies examining changes in digital reading configurations over time, the key finding of dynamic user preferences serves as a guiding principle. Researchers can utilize this understanding to adapt services dynamically in response to evolving user needs, ensuring sustained satisfaction in an ever-changing technological landscape.

Moreover, one notable limitation of our study is the lack of consideration for key variables such as the vendor of the digital reading product, the platform utilized, and the specific digital device employed by readers. These variables could potentially impact the digital reading experience and subsequent levels of reader service satisfaction. By not explicitly exploring these factors, our study may have overlooked important nuances that could contribute to a more comprehensive understanding of digital reading in college library settings. Future studies could investigate how factors such as the choice of digital reading platform, the features offered by different vendors, and the compatibility of digital devices influence reader perceptions of service quality and satisfaction within college libraries. Additionally, exploring how variations in digital reading experiences across different devices (e.g., computers, tablets) impact reader engagement and satisfaction levels could provide valuable insights for library practitioners aiming to enhance their digital offerings.

Another limitation relates to the complexity of human factors that influence reader service satisfaction. A central theme in our key findings was the acknowledgment of the complexity of human factors influencing reader service satisfaction. While we emphasized digital reading dimensions, the interplay with individual reading habits, preferences, and learning styles was not fully considered. This crucial insight informs the limitations and future research suggestions. Future studies should delve into the human-centered aspects of reader service satisfaction, aligning with our key finding that user preferences are diverse and flexible. By incorporating this understanding, researchers can unravel how personal preferences and learning styles interact with digital reading services, enriching the human dimension of library services.
